# Is the OJIP Test a Reliable Indicator of Winter Hardiness and Freezing Tolerance of Common Wheat and Triticale under Variable Winter Environments?

**DOI:** 10.1371/journal.pone.0134820

**Published:** 2015-07-31

**Authors:** Marcin Rapacz, Monika Sasal, Hazem M. Kalaji, Janusz Kościelniak

**Affiliations:** 1 Department of Plant Physiology, Faculty of Agriculture and Economics, University of Agriculture in Krakow, Krakow, Poland; 2 Department of Plant Physiology, Faculty of Agriculture and Biology, Warsaw University of Life Sciences, Warsaw, Poland; University of Hyderabad, INDIA

## Abstract

OJIP analysis, which explores changes in photosystem II (PSII) photochemical performance, has been used as a measure of plant susceptibility to stress. However, in the case of freezing tolerance and winter hardiness, which are highly environmentally variable, the use of this method can give ambiguous results depending on the species as well as the sampling year and time. To clarify this issue, we performed chlorophyll fluorescence measurements over three subsequent winters (2010/11, 2011/12 and 2012/13) on 220 accessions of common winter wheat and 139 accessions of winter triticale. After freezing, leaves were collected from cold-acclimated plants in the laboratory and field-grown plants. Observations of field survival in seven locations across Poland and measurements of freezing tolerance of the studied plants were also recorded. Our results confirm that the OJIP test is a reliable indicator of winter hardiness and freezing tolerance of common wheat and triticale under unstable winter environments. Regardless of species, the testing conditions giving the most reliable results were identical, and the reliability of the test could be easily checked by analysis of some relationships between OJIP-test parameters. We also found that triticale is more winter hardy and freezing tolerant than wheat. In addition, the two species were characterized by different patterns of photosynthetic apparatus acclimation to cold.

## Introduction

Freezing tolerance is a main component of winter hardiness, a trait that limits plant production in higher latitudes. Both traits are strongly influenced by environmental effects, which is apparently crucial for crop production when faced by climate change [1]. To monitor environmental effects on plant growth and development, chlorophyll fluorescence measurements have been used to study changes in photosynthetic activity triggered by environmental stresses such as nutrient deficiency [2], heavy metal soil contamination [3], salinity [4, 5], drought [6], light stress [7] and high [8–10] and low [11, 12] temperatures. The chlorophyll fluorescence measurement data can be analyzed by the so-called OJIP test, which is based on the theory of energy flow in thylakoid membranes, thereby facilitating a better understanding of the relationships between the biophysical side of photosynthesis and fluorescence signals [13, 14]. The OJIP test also provides information about the probability of the fate of absorbed light energy as well as detailed information on photosynthetic apparatus structure and function [8]. Recent studies of photosynthetic apparatus response to freezing based on the OJIP test have shown that the chlorophyll fluorescence techniques used to predict whole-plant freezing tolerance and, ultimately, winter hardiness are advantageous, but also have interesting limitations that make the reliability of the results dependent on certain conditions [15–17]. Although cold-induced changes in the photosynthetic apparatus are necessary for whole-plant cold acclimation [18, 19], freezing tolerance in the field depends on many factors based on complex environmental responses [20]. This complexity makes the results of chlorophyll fluorescence-based studies more difficult to understand, but can provide a unique opportunity to distinguish the effects of different environmental factors on freezing tolerance in the field [17].

Previous studies conducted on common wheat (*Triticum aestivum* L.) or triticale (×*Triticosecale* Wittm.), the most widely cultivated winter cereals in Poland, indicated that not all OJIP-test parameters were related to the freezing-tolerance level of the investigated plants [15, 17, 21, 22]. In addition, the usefulness of a given parameter seemed to depend on environmental conditions before sampling, freezing temperature or even plant species. The conclusion drawn was that the plants should be grown under field conditions during winter, with the leaves additionally frozen before measurements, for reliable estimation [15, 17]. Nevertheless, the biological basis of this phenomenon remains unsolved and the reliability of the results, which depend on environmental conditions, are unpredictable unless parallel tests of whole-plant freezing tolerance are performed. This inability to check the quality of the OJIP-analysis assessment without reliance on the traditional freezing test limits the use of chlorophyll-fluorescence studies of freezing tolerance to a supplementary role in freezing tolerance assessments under variable winter environments.

We hypothesize that differences in relationships between specific energy flux parameters in photosystem II (PSII) triggered by changing winter environments may affect the reliability of OJIP test-based studies of freezing tolerance. To verify our hypothesis, we collected leaves from cold-acclimated plants of common winter wheat and winter triticale grown in the laboratory and from field-grown plants over three winters. We performed chlorophyll fluorescence measurements on the collected leaves before and after freezing, and carried out a parallel control experiment to assess field survival and freezing tolerance of the studied plants.

## Materials and Methods

No specific permissions were required for our field experiments. The experiments were performed in the experimental fields owned by our University and plant breeding companies involved in the projects. The field studies did not involve endangered or protected species. The experiments were performed on two cereal species: common wheat (*Triticum aestivum L*.) and triticale (×*Triticosecale* Wittmack).

### Plant materials

The experiments were conducted during three subsequent winters: 2010/11, 2011/12 and 2012/13. During each winter, different sets of winter wheat and winter triticale advanced breeding lines (candivars) and common standards were evaluated for their winter survival and freezing tolerance. The standard cultivars were common wheat KWS Ozon, Muszelka and Tonacja and triticale Borwo and Fredro. The total number of wheat accessions used in this study was 69 (2010/11), 76 (2011/12) and 75 (2012/13), while the number of triticale accessions was 36 (2010/11), 54 (2011/12) and 49 (2012/13). Candivars were developed by five Polish breeding companies: Danko Plant Breeding (Choryń, Poland)—wheat (including standard Muszelka) and triticale (including standard Fredro); Smolice Plant Breeding (Smolice, Poland)—wheat and triticale; Strzelce Plant Breeding (Strzelce, Poland)—wheat (including standard Tonacja) and triticale (including standard Borwo); Małopolska Plant Breeding (Kraków, Poland)—wheat; and Poznań Plant Breeders (Tulce, Poland)—wheat. KWS Ozon is a cultivar bred by KWS Lochow GMBH (Bergen, Germany).

### Field assessment of winter hardiness

**Fig 1:**
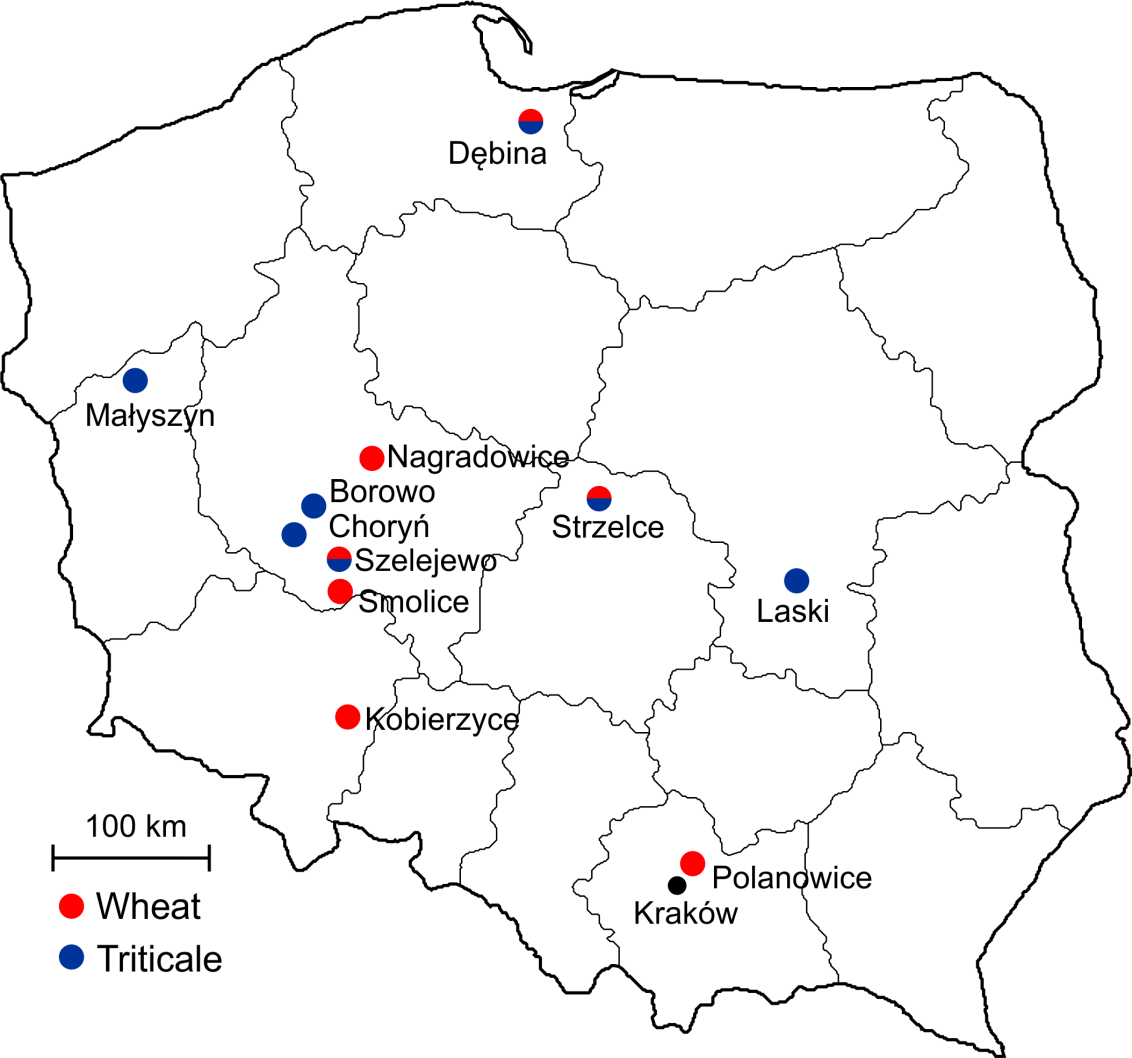
Experimental sites of field survival studies in Poland.

To assess winter hardiness, plants were sown in four replications (blocks) in 10-m^2^ plots containing 400 seeds per m^2^, with full randomization inside each block. The locations of experimental sites are shown in Fig 1. The experiments with wheat were set up at seven experimental sites: Dębina (N 54.130323, E 19.032393), Kobierzyce (N 50.972848, E 16.947629), Nagradowice (N 52.317566, E 17.145104), Polanowice (N 50.202783, E 20.084715), Smolice (N 51.698700, E 17.184260), Strzelce (N 52.317495, E 19.404706) and Szelejewo (N 51.858932, E 17.159140). The experiments with triticale were also performed at seven sites: Dębina, Strzelce and Szelejewo described above, and Borowo (N 52.123139, E 16.809329), Choryń (N 52.035333, E 16.770542), Laski (N 51.806330, E 21.133938) and Małyszyn (N 52.744548, E 15.173799).

Plant winter survival was assessed in the spring after vegetation emergence using a 1 to 9 score [23] as follows: 1 (100% winter-killed plants), 2 (>85% and <100% winter-killed plants), 3 (>70 and ≤85%), 4 (>55 and ≤70%), 5 (>40 and ≤55%), 6 (>25 and ≤40%), 7 (>10 and ≤25%), 8 (>0 and ≤10%) and 9 (plants with no visible damage).

### Freezing tolerance assessment using a field-laboratory method

To assess freezing tolerance, we used the modified field-laboratory method by Koch and Lehman [24] described in detail previously [15, 17]. Freezing tolerance was tested in Kraków (N 50.069014, E 19.845528) during two periods in the winters of 2010/11, 2011/12 and 2012/13. Plants were sown at the beginning of October in plastic boxes filled with a 1:1:1 (v/v/v) mixture of sand, clay soil and peat. Each accession was sown in six replicates in rows comprising 12 seeds, randomized in separate boxes. The boxes were then placed in the experimental field in blocks each containing one replication with full randomization inside each block. Weather conditions in the experimental fields were monitored with an electronic weather station. The boxes were transferred to a freezing chamber set at 0°C on the following dates: 3 December 2010, 25 February 2011, 20 January 2012, 10 February 2012, 18 January 2013 and 22 February 2012. The temperature was lowered by 1.5°C/h to –15°C, and the plants were frozen for 14 h in darkness. The temperature was then raised at a rate of 1.5°C/h. After reaching +2°C, the boxes were transferred to an unheated glasshouse maintained at 10–15°C and the plants were cut 1.5 cm above the soil level. After 3 weeks of growth, the percentage of re-growing plants was counted.

### Laboratory-based freezing tolerance assessment

Standard cultivars were assessed for freezing tolerance in a laboratory experiment set up in the same manner as described for the field-laboratory method. Two independent experiments were performed in 2010 and 2011 and the results were averaged. In this method, plants were cold-acclimated under a controlled environment for 24 days under the following conditions: 4/2°C day/night temperature, 8-h/16-h photoperiod and a light intensity of 200 μmol m^–2^ s^–1^ under HPS ‘Agro’ lamps (Philips, Brussels, Belgium). Cold acclimation began in the middle of November when the field temperature was below 10°C. After cold acclimation, plants were frozen and their regrowth was estimated as described for the field-laboratory method.

### Chlorophyll fluorescence measurements

Immediately before transferring plants to a freezer, 10 leaves (2–3 from each box) with no visible damage were collected from each accession. The leaves were placed in polyethylene bags with a string closure and ice was added to ensure nucleation. The samples were subjected to freeze-thawing in a programmed freezer in the dark. The temperature was decreased from 0°C to –18°C at a rate of 1.5°C/h and maintained at the final temperature for 4 h. The temperature was then increased at a rate of 1.5°C/h to +2°C. The leaves were stored at this final temperature until measurement. In the case of standard cultivars, chlorophyll fluorescence measurements were additionally performed without subjecting the leaves to freeze-thawing. Instead, the leaf samples were stored in +2°C during this time. Before measurement, leaves were transferred to room temperature conditions and dark-adapted using leaf clips (Hansatech, Kings Lynn, UK) for 30 min. Chlorophyll-a fluorescence transients were recorded using a Handy PEA fluorometer (Hansatech). For the measurements, saturating light pulse intensity was set to 3000 μmol m^–2^ s^–1^ with a pulse duration to 0.3 s.

Using the generated induction curve, we calculated various parameters of PSII performance [25, 26]. In particular, we calculated the following specific energy fluxes for single PSII reaction centers (RCs): absorbed energy flux (ABS/RC), trapped energy flux (TR_o_/RC), electron transport flux (ET_o_/RC) and dissipated energy flux (DI_o_/RC). Phenomenological energy fluxes calculated for the area of the photosynthetic sample (CS) at *t* = 0 were: absorbed energy flux per CS (ABS/CS), trapped energy flux per CS (TR_o_/CS), electron transport flux per CS (ET_o_/CS) and dissipated energy flux per CS (DI_o_/CS). Calculated PSII performance indexes and reaction center densities were as follows: performance index in relaxed and excited states (PI_CSo_ and PI_CSm_, respectively) on a CS basis (PI_CS_), performance index calculated on an absorption basis (PI_ABS_) and densities of Q_A_^-^ reducing PSII reaction centers at *t* = 0 and *t*_max_ (time to reach maximum fluorescence F_m_), corresponding to RC/CS_o_ and RC/CS_m_, respectively. Finally, we calculated the following yield ratios: the probability (at *t* = 0) of electron transport beyond Q_A_^-^ (ψ_o_), quantum yield of electron transport (at *t* = 0) (φ_Eo_) and maximum quantum yield of primary photochemistry (at *t* = 0) (φ_Po_ or F_v_/F_m_). Detailed calculations for OJIP parameters are shown in S1 Text.

### Statistical treatment

Both field and field-laboratory experiments were established using fully randomized block designs. Data were analyzed with Statistica 10.0 PL software (Statsoft, Tulsa, OK, USA). Normal distribution of the data was checked using histograms and analyzed with a Shapiro-Wilk W test. Data not following a normal distribution were arcsin square-root transformed prior to further processing. Statistical significance (P = 0.05) of factorial effects were tested with multi-factor ANOVA in General Linear Model, with accession (species or candivar/cultivar) and environment (year, location and sampling date) as factors. The Significance of Differences) between means was estimated with Tukey’s HSD (Honest significant difference test. Linear correlation coefficients (Pearson’s) were calculated between traits on the basis of means for single accessions. Principal component analysis (PCA) was used to analyze the data pattern and classification of standard cultivars. The PCA was performed by eigenvalue decomposition of a data correlation matrix.

## Results

Weather variations were responsible for different plant cold acclimation conditions during the three subsequent years of the experiment (Table 1). The onset of winter 2010/11 was relatively early. After a warm autumn (in October and November), the temperature dropped rapidly in December. Although such a sudden temperature reduction can result in insufficient levels of plant cold acclimation, the plants were not seriously damaged because they were blanketed with a thick snow cover before freezing. In the following months, freezing days without snow cover led to a lower survival rate by the time of the second sampling (Table 2). In all the field-locations no winter damages of triticale were observed during winter 2010/11, while survival rate of wheat were slightly variable between locations but also relatively high (Table 3). The autumn prior to winter 2011/12 was characterized by a gradual decrease in temperatures, thus allowing plants to become thoroughly cold acclimated (Table 1). The lowest temperatures were recorded in January, which included 2 days without snow cover, and in February, when snow cover existed in Kraków (Table 1) but not in the experimental fields in western Poland [17]. During that winter big differences in winter survival of both wheat and triticale were observed between field locations (Table 3). In some of them survival rate was very low. On the other hand correlation coefficients between field survival and freezing tolerance were high, especially in the case of wheat. The final winter (2012/13) was associated with either good conditions for cold acclimation (a gradual temperature decrease in the autumn) as well as good field-survival conditions (deep and long-lasting snow cover and minimum temperatures close to −17°C). This affects relatively high freezing tolerance in field-laboratory method and total overwintering of both species in the experimental fields (Tables 2 and 3). Freezing tolerance as assessed by the field-laboratory method was always higher in triticale than in common wheat (Table 2). Both cereals showed similar freezing tolerances only at the first sampling point during winter 2010/11. Triticale also displayed higher winter hardiness than wheat as assessed by the field-survival method (Table 3).

**Table 1:** Summary of meteorological data recorded during the experiment performed in Kraków.

Year	Month	Daily average temp.(°C)	Max. temp.(°C)	Min. temp.(°C)	Freezing days^a^	Days with sub-freezing^b^	Precipitation (mm)	Days with snowcover	Max. snowcover depth (mm)	Number of freezing days without snow cover
**2010**	**Oct.**	5.1	17.7	-3.8	0	14	11.5	0	0	0
	**Nov.**	6.4	20.4	-9.8	5	10	47.6	4	150	2
	**Dec.**	-5.4	5.4	-20.2	26	29	35.3	31	260	0
**2011**	**Jan.**	-1.2	8.7	-16.9	19	27	26.2	25	80	8
	**Feb.**	-2.6	9.6	-11.4	19	25	8.6	13	40	9
	**Mar**	3.2	18.7	-9.2	7	21	15.3	0	0	7
**2011**	**Oct.**	8.3	24	-4.4	0	6	30.3	0	0	0
	**Nov.**	2.3	16.8	-7	9	23	0.2	0	0	8
	**Dec.**	1.6	11.7	-12	7	26	36.9	3	30	0
**2012**	**Jan.**	-1.0	12.6	-16.7	13	22	51.2	22	110	2
	**Feb.**	-7.2	9.1	-23.3	21	26	26.3	24	190	0
	**Mar.**	4.6	21.8	-9.9	5	19	21.1	1	0	0
**2012**	**Oct.**	8.7	23.1	-6.2	2	7	96.9	4	50	0
	**Nov.**	5.0	17.3	-4.5	2	8	22.2	0	0	0
	**Dec.**	-2.8	8.5	-16.8	21	26	27.6	21	100	2
**2013**	**Jan.**	-2.5	10.3	-11.4	22	28	61.9	25	160	0
	**Feb.**	-0.4	6.4	-10.9	15	20	22.2	26	130	1
	**Mar.**	-0.9	15.2	-15.9	16	30	31.9	19	110	3

For detailed field temperatures, see [17].

^a^Days with mean temperature >0°C

^b^Days with minimum temperature <0°C

**Table 2:** Freezing tolerance of studied wheat and triticale accessions in field-laboratory studies (% survival after freezing).

Sampling date	Winter					
	2010/11		2011/12		2012/13	
	Wheat	Triticale	Wheat	Triticale	Wheat	Triticale
**1** ^**st**^ **(early winter)**	75.0^a^	67.3^a^	30.3^b^	42.0^a^	37.1^c^	77.1^a^
**2** ^**nd**^ **(late winter)**	9.5^d^	32.9^c^	15.0^d^	23.0^c^	33.1^c^	55.1^b^
**Mean**	44.4^**^	50.1^**^	23.4^**^	29.9^**^	35.1^***^	66.0^***^

Letters indicate homogeneity groups calculated separately for each trait and winter at *P* = 0.05 (Tukey’s HSD test).

*, **, *** after a mean value denote statistically significant differences between wheat and triticale at *P* = 0.05, 0.01 and 0.001, respectively.

**Table 3:** Winter hardiness of studied wheat and triticale accessions together with linear correlation coefficients with mean freezing tolerance recorded in field-laboratory studies as plant survival after freezing.

Location	Winter					
	2010/11		2011/12		2012/13	
	Wheat	Triticale	Wheat	Triticale	Wheat	Triticale
**Dębina**	6.37^c^ (0.66)	9.0^a^ (n.a.)	4.73^cd^	6.17^b^ (0.67)	9.0 (n.a.)	9.0 (n.a.)
**Strzelce**	6.57^c^ (0.32)	9.0^a^ (n.a.)	4.18^d^ (0.80)	2.64^f^ (0.59)	9.0 (n.a.)	9.0 (n.a.)
**Szelejewo**	8.86^a^	9.0^a^ (n.a.)	3.67^e^ (0.82)	5.65^bc^ (0.60)	9.0 (n.a.)	9.0 (n.a.)
**Kobierzyce**	8.73^a^ (0.34)	-	4.27^d^ (0.83)	-	9.0 (n.a.)	-
**Borowo**	-	9.0^a^ (n.a.)	-	3.91^d^ (0.66)	-	9.0 (n.a.)
**Nagradowice**	8.49^a^ (0.36)	-	2.52^f^ (0.68)	-	9.0 (n.a.)	-
**Choryń**	-	9.0^a^ (n.a.)	-	3.84^de^ (0.73)	-	9.0 (n.a.)
**Polanowice**	6.58^c^ (0.40)	-	3.42^e^ (0.86)	-	9.0 (n.a.)	-
**Laski**	-	9.0^a^ (n.a.)	-	5.01^c^ (0.66)	-	9.0 (n.a.)
**Smolice**	8.49^a^ (0.36)	-	6.45^b^ (0.83)	-	9.0 (n.a.)	-
**Małyszyn**	-	9.0^a^ (n.a.)	-	8.06^a^ (0.64)	-	9.0 (n.a.)
**Mean**	7.73^**^ (0.57)	9.0^**^ (n.a.)	4.18^**^ (0.86)	5.04^**^ (0.75)	9.0 (n.a.)	9.0 (n.a.)

Correlation coefficients shown in the table in parentheses after winter hardiness data are statistically significant at *P* = 0.05 (n.a.—not applicable because of the homogeneity of the winter hardiness values). Letters indicate homogeneity groups calculated separately for each trait and winter at *P* = 0.05 (Tukey’s HSD test).

*, **, *** after a mean value denote statistically significant differences between wheat and triticale at *P* = 0.05, 0.01 and 0.001, respectively.

Three wheat and two triticale accessions (standards) were studied over three winters. Differences in winter hardiness among wheat and triticale standards were only observed during the winter of 2011/12, with wheat and triticale also differentiated in the winter of 2010/11 (Table 4). The most winter-hardy wheat cultivar, Tonacja, also exhibited the highest survival rates according to field-laboratory experiments in 2010/11 and 2012/13. The cultivar Muszelka showed higher freezing tolerance than that of KWS Ozon, both in the laboratory as well as in 2010/11 and 2011/12 field-laboratory experiments (Table 4), but its field survival during the winter of 2011/12 was lower than that of KWS Ozon. In triticale, the Fredro cultivar was less (or equally) freezing tolerant and winter hardy than Borwo, regardless of the method or year of assessment.

**Table 4:** Winter hardiness (field-survival) and freezing tolerance (% survival in field-laboratory and laboratory experiments) of reference cultivars.

Cultivar	Field survival (1–9)			Field-laboratory experiment(% survival)			Laboratory experiment (% survival)
	2010/11	2011/12	2012/13	2010/11	2011/12	2012/13	Mean
**Tonacja (wheat)**	8.2^ab^	4.29^c^	9.0^a^	47.3^c^	12.3^de^	62.0^b^	80.0^b^
**Muszelka (wheat)**	7.9^b^	1.52^f^	9.0^a^	26.0^d^	10.2^e^	23.8^d^	70.1^c^
**KWS Ozon (wheat)**	8.0^b^	2.48^e^	9.0^a^	9.7^e^	0.50^f^	18.4^d^	59.9^d^
**Borwo (triticale)**	9.0^a^	5.21^c^	9.0^a^	61.2^b^	40.9^c^	73.8^a^	100.0^a^
**Fredro (triticale)**	9.0^a^	3.43^d^	9.0^a^	58.9^b^	22.5^d^	67.3^ab^	81.3^b^

Letters indicate homogeneity groups calculated separately for each trait (*P* = 0.05, Tukey’s HSD test).

Measurements of chlorophyll fluorescence parameters were obtained using leaves collected from field-grown plants and frozen under controlled conditions. Values of all OJIP-test parameters calculated in this study clearly differed among studied accessions regardless of the winter and date of measurements (S1 Table). The higher photosynthetic performance of the more freezing-tolerant triticale compared with wheat was observed only on the second sampling date during the winters of 2010/11 and 2011/12. The results of the OJIP analysis for all studied accessions were compared with data on plant survival after freezing and winter hardiness (Tables 5 and 6). This comparison indicated that the reliability of the chlorophyll fluorescence assessment of freezing tolerance depended on the winter and the sampling term, with a clear interaction between winter and species evident. Examples of averaged OJIP curves were given as supplemental materials (S1 Fig). In both species, correlations were higher when the chlorophyll fluorescence measurements were taken in the second term (in late winter). No significant correlations were observed between OJIP-test parameters measured in leaves sampled from laboratory-acclimated plants and data on freezing tolerance and winter hardiness. The highest correlations with freezing tolerance and winter hardiness for triticale and the lowest for wheat were observed during the winter of 2011/12. The highest correlations for common wheat were recorded in the winter of 2012/13. The same OJIP-test parameters generally showed the highest correlations with both winter hardiness and freezing tolerance regardless of sampling date and species. In common wheat, the highest correlations of OJIP-test parameters with plant survival after freezing, *r* = ~0.7, were observed during the winter of 2012/13, with ET_o_/CS, RC/CS_o_, RC/CS_m_ and TR_o_/CS being the best correlated parameters (Table 5). The same parameters, together with ET_o_/RC, were also the best indicators for prediction of winter field survival during the winter of 2010/11. During that winter, ET_o_/RC and ET_o_/CS were the parameters having the highest correlations with plant survival after freezing. On the other hand, during the winter of 2011/12, when correlation coefficients between OJIP-test parameters and freezing tolerance and winter survival were lower than in other winters (*r* = ~0.4), ET_o_/CS, RC/CS_o_, RC/CS_m_ and TR_o_/CS exhibited coefficients similar to those of the PSII quantum yields φ_Po_ and φ_Eo_.

**Table 5:** Linear correlation coefficients between OJIP-test parameters and freezing tolerance—FT (plant survival after freezing) and winter hardiness—WH (field survival) recorded in common winter wheat during three studied winters.

OJIP-test parameter	Winter (sampling period for chlorophyll fluorescence studies)									
	2010/11 (1)		2010/11 (2)		2011/12 (1)		2011/12 (2)		2012/13 (1)	2012/13 (2)
	FT	WH	FT	WH	FT	WH	FT	WH	FT	FT
**φ**_**Po**_	0.28	0.30	0.35	0.56			0.40	0.47	0.26	0.65
**ABS/RC**	-0.39	-0.31						-0.28		-0.52
**ψ**_**o**_			0.47	0.59	0.29	0.30	0.31			0.65
**φE**_**o**_		0.25	0.40	0.54	0.27	0.29	0.44	0.47		0.67
**PI**_**CSo**_	0.30	0.30	0.37	0.49	0.28	0.31	0.42	0.36	0.41	0.69
**PI**_**CSm**_	0.31	0.31	0.32	0.43	0.28	0.30	0.40	0.33	0.44	0.66
**PI**_**ABS**_	0.31	0.30	0.31	0.44	0.25	0.28	0.45	0.39	0.26	0.64
**ABS/CS**				-0.47					0.26	
**TR**_**o**_ **/CS**			0.48	0.61			0.35	0.42	0.34	0.69
**ET**_**o**_ **/CS**			0.50	0.59	0.28	0.26	0.40	0.43	0.41	0.73
**DI**_**o**_ **/CS**			-0.26	-0.55			-0.31	-0.31		-0.52
**RC/CS**_**o**_			0.45	0.62			0.41	0.44	0.42	0.70
**RC/CS**_**m**_	0.30	0.30	0.39	0.56			0.41	0.40	0.53	0.71
**ET**_**o**_ **/RC**			0.54	0.61	0.32	0.25				0.68
**TR**_**o**_ **/RC**	-0.33		0.39	0.52				-0.27		-0.52
**DI**_**o**_ **/RC**	-0.37	-0.36		-0.48				-0.28		-0.26

Sampling periods were early winter (1) and late winter (2). All presented values are statistically significant at *P* = 0.05. In the winter of 2012/13, field survival was the same for all accessions.

**Table 6:** Linear correlation coefficients between OJIP-test parameters and freezing tolerance—FT (plant survival after freezing) and winter hardiness—WH (field survival) recorded in winter triticale during three studied winters.

OJIP-test parameter	Winter (sampling period for chlorophyll fluorescence studies)							
	2010/11 (1)	2010/11 (2)	2011/12 (1)		2011/12 (2)		2012/13 (1)	2012/13 (2)
	FT	FT	FT	WH	FT	WH	FT	FT
**φ**_**Po**_			0.32	0.36	0.59	0.77		0.47
**ABS/RC**						-0.39		-0.37
**ψ**_**o**_							0.34	
**φE**_**o**_					0.57	0.76		0.39
**PI**_**CSo**_					0.58	0.76		0.35
**PI**_**CSm**_					0.57	0.73		
**PI**_**ABS**_					0.50	0.70	0.32	0.35
**ABS/CS**		0.48			0.55	0.61		
**TR**_**o**_ **/CS**		0.46			0.65	0.81		0.46
**ET**_**o**_ **/CS**		0.44			0.64	0.81		0.40
**DI**_**o**_ **/CS**						-0.45		-0.35
**RC/CS**_**o**_		0.44			0.64	0.80		0.57
**RC/CS**_**m**_					0.63	0.80		0.51
**ET**_**o**_ **/RC**								
**TR**_**o**_ **/RC**								-0.36
**DI**_**o**_ **/RC**						-0.39	-0.34	

Sampling periods were early winter (1) and late winter (2). All presented values are statistically significant at *P* = 0.05. In the winters of 2010/11 and 2012/13, field survival was the same for all accessions.

In triticale, the highest correlations between OJIP-test parameters and freezing tolerance were observed during the winter of 2011/12 (Table 6). During this winter, a very high correlation (>0.8) with winter survival data was also observed. The best parameters for predicting either winter survival or freezing tolerance were the same as in wheat during the winter of 2012/13, namely, TR_o_/CS, ET_o_/CS, RC/CS_o_ and RC/CS_m_. RC/CS_o_ was also clearly the best parameter for the estimation of freezing tolerance during the winter of 2012/13, and, together with ET_o_/CS, TR_o_/CS and ABS/CS, constituted the only ones significantly correlated with plant freezing tolerance during the winter of 2010/11.

Also noteworthy is that negative correlations were sometimes found between ABS/RC and TR_o_/RC and both freezing tolerance and winter hardiness, indicating a better photosynthetic performance of the single active PSII reaction center in the less-tolerant accessions (Tables 5 and 6). A similar effect was observed during the winter of 2010/11 in wheat, where winter survival was negatively correlated with energy absorption by leaf cross section (ABS/CS). Negative correlation coefficients were also computed in the case of DI_o_/RC and DI_o_/CS, but the values of these parameters increased with increasing levels of PSII damage.

**Fig 2:**
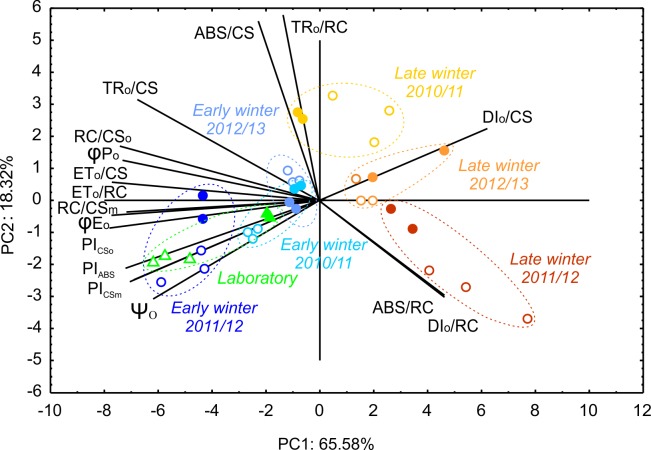
Results of principal component analysis showing two major components for different sampling dates and locations for triticale (closed symbols) and wheat (open symbols) standard cultivars. Data from field and laboratory cold-acclimated plants, indicated by circles and triangles, respectively, represent means from two independent experiments.

We performed a detailed analysis of the effects of freezing on photochemical performance of wheat and triticale standard cultivars during different winters and sampling periods. PCA (Fig 2, S2 Table) suggested that the main factor affecting relationships between different OJIP-test parameters was the environmental conditions before sampling. All late-winter-measured data points were placed on the right side of the PCA plot, whereas both early-winter and laboratory-sampled data appeared on the left. Interestingly, most of the OJIP-test parameters were responsible for the variation seen between accessions in the left part of the graph, with only three parameters contributing to those on the right. ABS/RC and DI_o_/RC were responsible for observed differences in the late-winter sampling in 2011/12 (and DI_o_/CS also in the case of late winter sampling in 2012/13); in addition, these two parameters, together with TR_o_/RC and ABS/CS, were the main source of variation in late winter in 2010/11. Measurement year also had an important effect on late-winter measurements, as its contributions to principal components 1 and 2 (PC1 and PC2) respectively increased or decreased according to winter severity. Among early-winter samplings, the results obtained during the winter of 2011/12, when plants were damaged in the field before early-winter measurements, were distinct from those of the remaining winters. In the case of wheat, the results were very similar to those of the laboratory sampling. Differences between species were evident regardless of winter and measurement date, with one exception—less freezing-tolerant triticale cultivar Fredro, which performed similarly to wheat genotypes in 2012/13. We also found it remarkable that results of the OJIP analysis highly correlated with freezing tolerance and field survival, namely, the second sampling date during the winter of 2011/12 in the case of triticale and the second sampling date in 2012/13 for common wheat, had very similar positions on the PCA plot, with PC2 close to 0 and PC1 with a low, positive value.

To compare PSII photochemical performance during freezing tests performed on different sampling dates, correlations between OJIP-test parameters were separately calculated for each measurement scheme (laboratory and field-laboratory methods over three winters and two sampling periods) (S3 Table). Different patterns of relationships were observed between OJIP-test parameters depending on whether or not the results of chlorophyll fluorescence studies were highly correlated with freezing tolerance or winter hardiness (Table 7). For both species, samplings during late winter, which gave more reliable results than in early winter, were characterized by statistically significant, negative correlations between DI_o_/CS and the parameters that may be considered as indirect indicators of freezing tolerance and winter hardiness (TR_o_/CS, ET_o_/CS, RC/CS_o_ and RC/CS_m_) (Table 6). It should also be noted that correlations between indirect indicators of freezing tolerance and DI_o_/CS in triticale were statistically insignificant in two cases during late-winter sampling in 2010/11, a period when very low correlations between chlorophyll fluorescence measurements and both freezing tolerance and field survival were observed. In addition, all reliable data sets showed positive correlations between ET_o_/RC and the potential freezing-tolerance indicators (TR_o_/CS, ET_o_/CS, RC/CS_o_ and RC/CS_m_) with the exception of winter wheat in late-winter 2011/12, when correlations between OJIP-test parameters and plant survival were low (Table 5).

**Table 7:** Linear correlation coefficients between selected OJIP-test parameters measured in winter wheat and triticale on different sampling times during three studied winters and in laboratory experiments (mean).

Winter (sampling) or laboratory	Species	Wheat				Triticale			
	OJIP-test parameter	TR_o_/CS	ET_o_/CS	RC/CS_o_	RC/CS_m_	TR_o_/CS	ET_o_/CS	RC/CS_o_	RC/CS_m_
2010/11 (1)	DI_o_/CS	0.601*	0.196	0.483*	0.020	0.592*	-0.337*	0.139	-0.662*
	ET_o_/RC	0.146	0.591*	-0.028	0.263*	-0.020	0.896*	0.050	0.506*
2010/11 (2)	DI_o_/CS	-0.543*	-0.459*	-0.564*	-0.481*	-0.274	-0.305	-0.432*	-0.496*
	ET_o_/RC	0.746*	0.842*	0.751*	0.773*	0.937*	0.980*	0.915*	0.791*
2011/12 (1)	DI_o_/CS	0.794*	0.106	0.327*	-0.520*	0.863*	0.551*	0.532*	-0.425*
	ET_o_/RC	0.008	0.690*	-0.176	0.268*	0.449*	0.727*	0.287*	0.212
2011/12 (2)	DI_o_/CS	-0.444*	-0.496*	-0.454*	-0.489*	-0.476*	-0.503*	-0.543*	-0.538*
	ET_o_/RC	-0.144	-0.096	-0.194	-0.142	0.158	0.163	0.160	0.170
2012/13 (1)	DI_o_/CS	0.573*	0.057	0.378*	-0.094	0.367*	0.117	0.223	-0.105
	ET_o_/RC	0.149	0.826*	0.287*	0.514*	0.206	0.641*	0.253	0.371*
2012/13 (2)	DI_o_/CS	-0.658*	-0.745*	-0.741*	-0.793*	-0.525*	-0.564*	-0.664*	-0.659*
	ET_o_/RC	0.852*	0.952*	0.884*	0.925*	0.815*	0.943*	0.725*	0.833*
Laboratory	DI_o_/CS	0.047	-0.475*	0.175	-0.699*	-0.157	-0.546*	-0.312	-0.744*
	ET_o_/RC	0.486*	0.902*	-0.218	0.586*	0.732*	0.949*	0.149	0.696*

Sampling times were early winter (1) and late winter (2). Values marked by an asterisk are statistically significant at *P* = 0.05.

**Fig 3:**
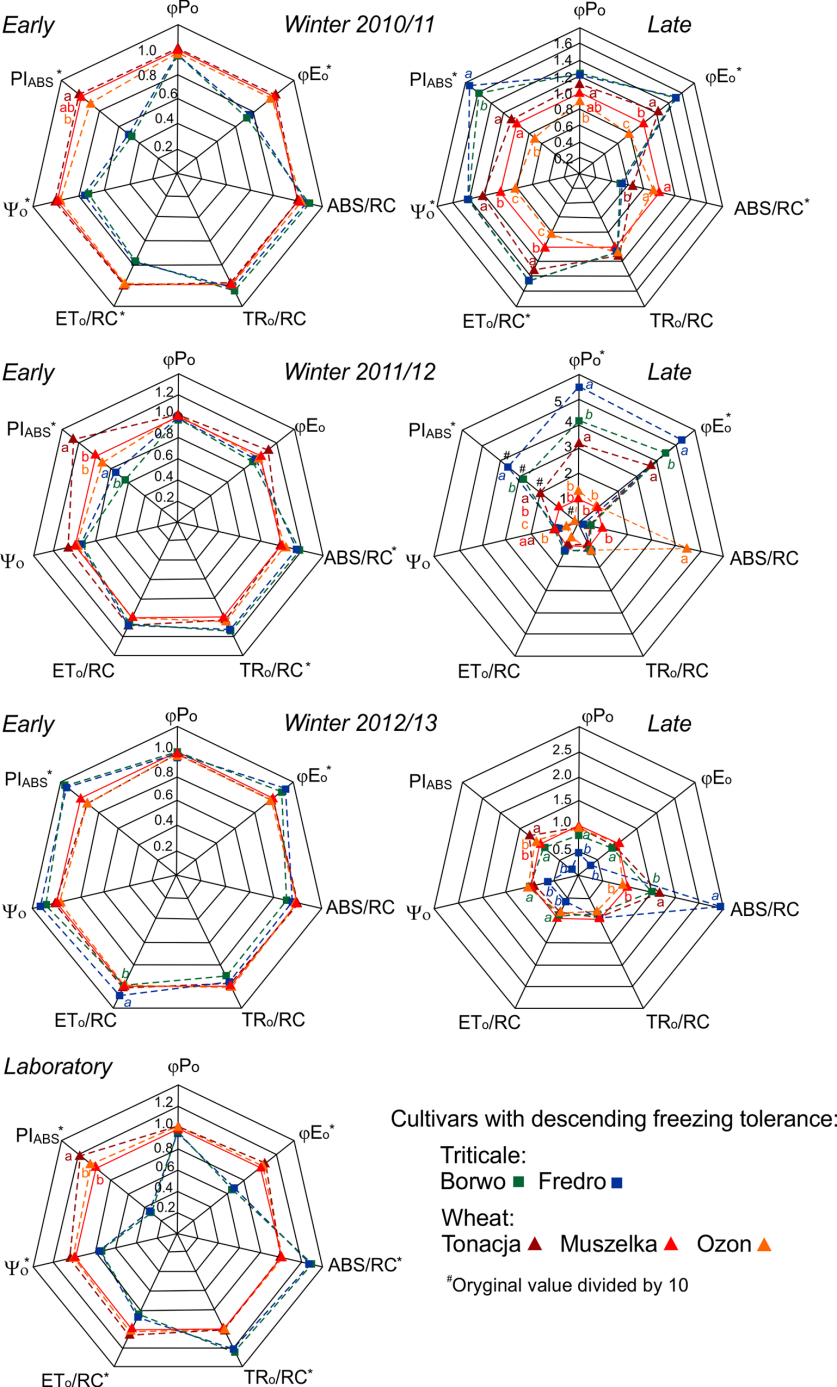
Energy transfer efficiencies (φPo, φEo and ψ_o_), overall performance index of photosystem II (PSII) (PI_ABS_) and specific energy fluxes in PSII (ABS/RC, TR_o_/RC and ET_o_/RC) for different sampling dates. Values shown are relative to results obtained from the medium winter-hardy wheat cultivar Muszelka (red line). Data for laboratory cold-acclimated plants are the means of two independent experiments. Values marked with the same letter denote the lack of statistical significance between cultivars of triticale (italicized) and wheat (*P* = 0.05, Tukey’s HSD test). Statistical significance of differences between species is indicated by asterisks outside the graphs (*P* = 0.05, *t*-test).

Cold acclimation under laboratory conditions resulted in clear differences in chlorophyll fluorescence parameters between species (Fig 3). Compared with wheat, triticale exhibited lower values of PI_ABS_, φ_Eo_, ψ_o_ and ET_o_/RC, but higher values of ABS/RC and TR_o_/RC. The only significant difference between cultivars within species was observed in the case of PI_ABS_ in wheat. Following either laboratory cold acclimation or early sampling in 2011/12, the freezing-tolerant cultivar Tonacja exhibited the highest value of PI_ABS_. Noteworthily, a very similar pattern of differences between species was observed in the case of first-sampling period data during the winters of 2010/11 and 2011/12, when hardening conditions were close to the optimum also simulated during laboratory cold acclimation (Fig 3, S1 Table). Laboratory and early-winter data from 2010/11 and 2011/12 also clustered together in the PCA plot (Fig 2). Interestingly, differences in the photochemical performance of wheat and triticale in late-winter 2010/11 and partly in 2011/12 were the opposite of those observed after laboratory or optimum field cold acclimation (i.e., lower values of PI_ABS_, φE_o_ and ψ_o_ in wheat; Fig 3, S1 Table). At the end of the winter of 2011/12, φ_po_ values were consistent with the freezing tolerance and field survival of the studied accessions (Fig 3, Table 4). During early-winter 2012/13, as in late-winter 2010/11 and 2011/12, higher values of PI_ABS_, φ_Eo_ and ψ_o_ were observed in triticale than in wheat. The less freezing-tolerant triticale cultivar Fredro when sampled in late-winter 2012/13 showed considerably lower PSII photochemical activity, other than TR_o_/CS and ABS/RC, compared with the remaining standards. During the same sampling period, lower TR_o_/RC values were observed in triticale than in wheat both before and after freezing of leaves (Fig 3, S1 Table).

## Discussion

Our previous studies suggested that chlorophyll fluorescence measurements performed on detached leaves followed by OJIP tests could serve as indicators of freezing tolerance and winter hardiness in triticale [15] and wheat [17]. Depending on plant growth conditions before sampling, however, different correlations were observed between measured chlorophyll fluorescence parameters and winter hardiness and/or plant freezing tolerance, thus calling into question the trustworthiness of the chlorophyll fluorescence data. In the present study, we confirmed that test reliability varied depending on plant growth conditions prior to testing, but also determined that data quality could be simply checked just by using OJIP data sets. As previously demonstrated and discussed [17], the chlorophyll fluorescence measurements should be performed on plants subjected to winter field conditions. Only such exposure allows the complex environmental factors to acclimate the photosynthetic apparatus to freezing tolerance levels equivalent to those observed for entire plants. Unfortunately, cold acclimation in the laboratory is not the best option for testing real winter hardiness, as shown previously not only in the case of chlorophyll fluorescence-based estimations [15, 17], but also in the case of plant survival tests [27].

From the present study, recommended parameters to be tested regardless of species are the number of active PSII reaction centers per leaf area—RC/CS_o_ and RC/CS_m_—and the energy fluxes for trapping and electron transport per excited leaf cross-section—TR_o_/CS and ET_o_/CS, respectively. To check for data reliability, a negative correlation should be observed between these parameters and energy flux for dissipation (DI_o_/CS). Such a correlation may indicate that energy dissipation in the leaf cross-section depends only on partial deactivation of PSII reaction centers, which cannot transfer energy upstream of PSII as a consequence of damage to thylakoid membranes. A positive relationship between the degree of thylakoid membrane damage and the reliability of chlorophyll fluorescence-based estimates of freezing tolerance has also been suggested earlier [15, 17]. It is possible that only field cold acclimation that includes some freezing episodes can acclimate thylakoid membranes to levels similar to those of the plasmalemma responsible for whole plant survival [28]. Interestingly, electron transport between primary quinon of PSII (*Q*_A_) and plastoquinol was also reported as the most susceptible to heat stress, which results in significant decrease in the number of active PSII reaction centers [9, 10]. In cases where leaves are heavily damaged in the field and thus parts of sampled leaves are senescent, OJIP-based indirect testing of freezing tolerance and winter hardiness may fail. When test parameters (RC/CS_o_, RC/CS_m_, TR_o_/CS and ET_o_/CS) are negatively correlated with DI_o_/CS, excessive PSII damage may be recognized by the lack of a positive correlation between test parameters and ET_o_/RC. This absence of a positive correlation may be the consequence of the stress induced damage of PSII reaction centers, which may increase energy flows observed in single active reaction center [29].

When the above-mentioned conditions are met, as shown in this study, some OJIP-analysis parameters are highly correlated with the results of multiple field survival studies or the results of field-laboratory plant survival tests. These correlation levels are similar to those observed between different locations and years in winter hardiness studies as well as between winter hardiness and freezing tolerance [15, 17, 27].

In other studies of freezing tolerance [16, 30] using an experimental protocol similar to the one in this study, F_v_/F_m_ (φ_Po_) was confirmed to be the chlorophyll fluorescence parameter most highly correlated with freezing tolerance. In the present study, φ_Po_ was well correlated (*r* > 0.6) with field survival/freezing tolerance only at the end of the winter of 2011/12 in triticale and at the end of the winter of 2012/13 in common wheat. At these time points, the freezing tolerance of triticale and wheat displayed the highest variation between cultivars due to winter conditions. In both species, F_v_/F_m_ has also been previously reported to not be the best parameter for freezing tolerance estimation [15, 17]. In the case of species with lower freezing tolerance than that of wheat and triticale, such as barley and durum wheat studied in [16], oats studied in [30], or *Arabidopsis thaliana* studied in [31] winter or even laboratory conditions were possibly sufficient to generate large variations in freezing tolerance between cultivars. As the result F_v_/F_m_ was reported there to be a good indicator of freezing tolerance. On the other hand critical reports about this parameter was published in the case of wheat, triticale, Scots pine and *Rhododendron ferrugineum* [15, 17, 21, 22, 32, 33]. All these species should be considered as having moderate to high freezing tolerance. Based on the results presented herein it can be concluded that the reason of non-successful use of F_v_/F_m_ as freezing tolerance indicator may be not-sufficient level of PSII reaction centers damages observed during experiments reported in the critical papers. The same was also suggested before in a study where F_v_/F_m_ was considered as good indicator of freezing tolerance in wheat only after photoinhibitory treatment performed during recovery after freezing [21].

In our study, common wheat and triticale appeared to undergo photosynthetic acclimation to cold via different mechanisms. This difference was especially visible during controlled cold acclimation, when the pattern of photosynthetic acclimation was not disturbed by other factors, especially snow cover, that considerably decrease light intensity in the field. No significant differences were observed in OJIP-test parameters between common wheat and triticale before cold treatment (data not shown). After cold treatment, and to some extent at the beginning of winter, triticale was found to exhibit lower PSII photochemical performance (lower PI_ABS_, φE_o_ and ψ_o_) along with higher light energy absorption and trapping in single reaction centers. Because no leaf damage or chlorophyll loss was observed during cold acclimation of triticale, this situation may have been due to increased energy dissipation. The observed effect, termed the non-photochemical mechanism of photosynthetic acclimation to cold, has been previously reported to be more highly expressed in more freezing-tolerant *Festuca pratensis* than in less freezing-tolerant *Lolium multiflorum* and to contribute to higher freezing tolerance in *Lolium* × *Festuca* hybrids [34, 35]. Nevertheless, speculation regarding extension of the non-photochemical mechanism of photosynthetic cold acclimation in rye, which is more freezing tolerant than wheat, to triticale, is difficult. Both rye and wheat have been previously reported to use a photochemical mechanism of photosynthetic acclimation consisting of increased photosynthetic capacity before winter [36]. The inferred expression of the non-photochemical mechanism in triticale may thus be the result of the unique combination of rye- and reduced wheat genomes. In the case of wheat, photochemical activity measured as PI_ABS_ in standard cultivars after controlled cold acclimation or in early winter was in accord with their freezing tolerance levels. This relationship has been previously seen in many species (including wheat) in which the photochemical mechanism of photosynthetic acclimation to cold is operative [18, 19, 36, 37].

At the end of each of the three winters, triticale was found to have a higher photosynthetic activity than wheat. This finding indicates lower levels of damage to the photosynthetic apparatus, possibly due to the more efficient photosynthetic cold acclimation (lower sensitivity to cold-induced photoinhibition) and higher winter hardiness of triticale (lower damage to entire plants). In the case of 2011/12, a particularly harsh winter, values of the photoinhibition-related parameter φ_po_ (F_v_/F_m_) were highest in the most freezing-tolerant cultivars of either species. A positive correlation between tolerance to cold-induced photoinhibition of photosynthesis and freezing tolerance has been shown previously, including in triticale and wheat [15, 18, 36, 38].

In this study, we directly compared the freezing tolerance of the photosynthetic apparatus and that of entire plants with winter hardiness in a wide range of common wheat and triticale accessions. In all tests, similar to an earlier report [39], triticale was superior to wheat. Different results were reported elsewhere, where hexaploid triticales, showed cold hardiness levels similar to their tetraploid wheat parents with the exception of less winter-hardy wheats; this observation suggests that the superior cold hardiness of the rye genome is suppressed in triticale [40]. The higher freezing tolerance and winter hardiness of triticale is thus instead a consequence of the unique gene composition and altered expression of the hybrid, as discussed earlier with respect to photosynthetic acclimation. In the cited study [40], however, the winter hardiness of triticale was not found to be superior to wheat. A possible explanation for this discrepancy is that in our experiment and the experiment reported in [39] that confirmed triticale’s superior winter hardiness, wheat and triticale advanced breeding lines and cultivars were used, whereas synthetic triticale hybrids, which were not selected for winter hardiness in breeding programs, were used in [40].

## Conclusions

The OJIP test may be a reliable indicator of winter hardiness and freezing tolerance of common wheat and triticale under variable winter environments. Before controlled freezing, leaves showing no substantial damage should be collected from fields in late winter. The best indirect measures of freezing tolerance are TR_o_/CS, ET_o_/CS, RC/CS_o_ and RC/CS_m_. The quality of the test results can be confirmed by analysis of some relationships between OJIP-test parameters. TR_o_/CS, ET_o_/CS, RC/CS_o_ and RC/CS_m_ values should be negatively correlated with those of DI_o_/CS, which indicates sufficient field adaptation. When this condition is fulfilled, the lack of positive correlations of TR_o_/CS, ET_o_/CS, RC/CS_o_ and RC/CS_m_ with ET_o_/RC indicates excessive levels of field damage.

In our study, common wheat and triticale were characterized by having different patterns of photosynthetic acclimation to cold and different freezing tolerances. Winter wheat, which is less freezing tolerant and less winter hardy, had higher photosynthetic activity after controlled cold acclimation and in early winter than triticale. During late winter, the photochemical activity of triticale is higher than that of wheat, indicating the lower sensitivity of triticale’s photosynthetic apparatus to winter conditions.

## Supporting Information

S1 Fig Averaged chlorophyll fluorescence induction curves recorded in wheat and triticale during winters and sampling times contrasting with the degree of cold acclimation and field damages of the leaves. Time-points used for OJIP-test parameters calculation were indicated.
(TIF)

S1 Table Mean, maximum and minimum OJIP-test parameters recorded in wheat and triticale accessions after freezing during different winters and sampling periods. Asterisks are used to denote mean values that are statistically significantly different between wheat and triticale at *P* = 0.05.
(DOCX)

S2 Table Contribution of changes in different OJIP test parameters to the total variation of principal components. (DOCX)

S3 Table Linear correlation coefficients between all calculated OJIP-test parameters at different sampling times in wheat (A) and triticale (B). Names of parameters recommended for freezing tolerance assessment are shown in green. Values of correlation coefficients in red are statistically significant at *P* = 0.05. Coefficients of correlation with DI_o_/CS and ET_o_/RC are marked in orange and yellow, respectively.
(XLSX)

S1 Text Calculations of OJIP parameters. (DOCX)

## References

[1] Rapacz M, Ergon Å, Höglind M, Jørgensen M, Jurczyk B, Østrem L, et al Overwintering of herbaceous plants in a changing climate—still more questions than answers. Plant Sci. 2014;225: 34–44. 10.1016/j.plantsci.2014.05.009 25017157

[2] Kalaji HM, Oukarroum A, Alexandrov V, Kouzmanova M, Brestic M, Zivcak M, et al Identification of nutrient deficiency in maize and tomato plants by *in vivo* chlorophyll *a* fluorescence measurements. Plant Physiol Bioch. 2014;81: 16–25. 10.1016/j.plaphy.2014.03.029 24811616

[3] Żurek G, Rybka K, Pogrzeba M, Krzyżak J, Prokopiuk K. Chlorophyll *a* Fluorescence in evaluation of the effect of heavy metal soil contamination on perennial grasses. PLoS ONE 2014;9: e91475 10.1371/journal.pone.0091475 24633293 PMC3954697

[4] Kalaji HM, Bosa K, Kościelniak J, Żuk-Gołaszewska K. Effects of salt stress on photosystem II efficiency and CO_2_ assimilation of two Syrian barley landraces. Environ Exp Bot. 2011a;73: 64–72.

[5] Yan K, Shao HB, Shao C, Chen P, Zhao S, Brestic M, et al Physiological adaptive mechanisms of plants grown in saline soil and implications for sustainable saline agriculture in coastal zone. Acta Physiol Plant. 2013;35: 2867–2878.

[6] Rapacz M, Kościelniak J, Jurczyk B, Adamska A, Wójcik M. Different patterns of physiological and molecular response to drought in seedlings of malt- and feed-type barleys (*Hordeum vulgare*). J Agron Crop Sci. 2010;196: 9–19.

[7] Kalaji HM, Carpentier R, Allakhverdiev SI, Bosa K. Fluorescence parameters as early indicators of light stress in barley. J Photoch Photobio B. 2012;112: 1–6. 10.1016/j.jphotobiol.2012.03.009 22561010

[8] Kalaji HM, Bosa K, Kościelniak J, Hossain Z. Chlorophyll a fluorescence—a useful tool for the early detection of temperature stress in spring barley (*Hordeum vulgare* L.). OMICS 2011b;15: 925–934. 22106950 10.1089/omi.2011.0070

[9] Yan K, Chen P, Shao HB, Zhao S. Characterization of photosynthetic electron transport chain in bioenergy crop Jerusalem artichoke (*Helianthus tuberosus* L.) under heat stress for sustainable cultivation Ind Crop Prod. 2013;50: 809–815.

[10] Yan K, Chen P, Shao HB, Shao C, Zhao S, Brestic M. Dissection of photosynthetic electron transport process in sweet sorghum under heat stress. PLoS One 2013;8: e62100 10.1371/journal.pone.0062100 23717388 PMC3663741

[11] Borawska-Jarmułowicz B, Mastalerczuk G, Pietkiewicz S, Kalaji MH. Low temperature and hardening effects on photosynthetic apparatus efficiency and survival of forage grass varieties. Plant Soil Env. 2014a;60: 177–183.

[12] Borawska-Jarmułowicz B, Mastalerczuk G, Kalaji HM, Carpentier R, Pietkiewicz S, Allakhverdiev SI. Photosynthetic efficiency and survival of *Dactylis glomerata* and *Lolium perenne* following low temperature stress. Russ J Plant Physl. 2014b; 61: 281–288.

[13] Strasser RJ, Tsimilli-Michael M, Srivastava A. Analysis of the chlorophyll a fluorescence transient In: Papageorgiou G, Govindjee, editors. Advances in photosynthesis and respiration. Chlorophyll a fluorescence: a signature of photosynthesis. Dordrecht: Springer; 2004 p. 321–362.

[14] Chernev P, Goltsev V, Strasser RJ. A highly restricted model approach quantifying structural and functional parameters of photosystem II probed by the chlorophyll a fluorescence rise. Ecol. Eng. Ecol. Protect. 2006;6: 19–29.

[15] Rapacz M, Sasal M, Gut M. Chlorophyll fluorescence—based studies of frost damage and the tolerance for cold-induced photoinhibition in freezing tolerance analysis of triticale (×*Triticosecale* Wittmack). J Agron Crop Sci. 2011;197: 378–389.

[16] Badeck F-W, Rizza F. A combined field/laboratory method for assessment of frost tolerance with freezing tests and chlorophyll fluorescence. Agronomy 2015;2015: 71–88.

[17] Rapacz M, Sasal M, Wójcik-Jagła M. Direct and indirect measurements of freezing tolerance: Advantages and limitations. Acta Physiol Plant. 2015 10.1007/s11738-015-1907-7

[18] Crosatti C, Rizza F, Badeck FW, Mazzucotelli E, Cattivelli L. Harden the chloroplast to protect the plant. Physiol Plant. 2013;147: 55–63. 10.1111/j.1399-3054.2012.01689.x 22938043

[19] Hüner NPA, Bode R, Dahal K, Busch FA, Possmayer M, Szyszka B, et al Shedding some light on cold acclimation, cold adaptation and phenotypic plasticity. Botany 2013;91: 127–136.

[20] Gusta LV, Wisniewski M. Understanding plant cold hardiness: an opinion. Physiol Plant. 2013;147: 4–14. 10.1111/j.1399-3054.2012.01611.x 22409670

[21] Rapacz M. Chlorophyll *a* fluorescence transient during freezing and recovery in winter wheat. Photosynthetica 2007;45: 409–418.

[22] Rapacz M, Woźniczka A. A selection tool for freezing tolerance in common wheat using the fast chlorophyll *a* fluorescence transient. Plant Breed. 2009;128: 227–234.

[23] Prášil IT, Prášilová P, Mařík P. Comparative study of direct and indirect evaluations of frost tolerance in barley. Field Crops Res. 2007;102:1–8.

[24] Koch MD, Lehman EO. Resistenzeigeschatten im Gärsten und Weizensortiment Gatersleben, 7 Prüfung der Frostresistenzpflanze. D.A.I. 1969;XIV: 263–282.

[25] Strasser RJ, Srivastava A, Govindjee. Polyphasic chlorophyll *a* fluorescence transient in plants and cyanobacteria. Photochem. Photobiol. 1995;61: 32–42.

[26] Strasser RJ, Srivatava A, Tsimilli-Michael M. The fluorescence transient as tool to characterize and screen photosynthetics samples In: Yunus M, Pathre U, Mohanty P, editors. Probing photosynthesis: mechanism, regulation and adaptation. Bristol: Taylor and Francis; 2000 p. 445–483.

[27] Gusta LV, O’Connor BJ, Gao J-P, Jana S. A re-evaluation of controlled freeze-tests and controlled environment hardening conditions to estimate the winter survival potential of hardy winter wheats. Can J Plant Sci. 2001;81: 241–246.

[28] Steponkus PL. Role of the plasma membrane in freezing injury and cold acclimation. Ann Rev Plant Physiol. 1984;35: 543–584.

[29] Soja G, Pfeifer U, Soja AM. Photosynthetic parameters as early indicators of ozone injury in apple leaves. Physiol Plant. 1998;104: 639–645.

[30] Rizza F, Pagani D, Stanca AM, Cattivelli L. Use of chlorophyll fluorescence to evaluate the cold acclimation and freezing tolerance of winter and spring oats. Plant Breed. 2001;120: 389–396.

[31] Mishra A, Mishra KB, Höermiller II, Heyer AG, Nedbal L. Chlorophyll fluorescence emission as a reporter on cold tolerance in *Arabidopsis thaliana* accessions. Plant Signal Behav. 2011;6: 301–310. 21427532 10.4161/psb.6.2.15278 PMC3121992

[32] Neuner G, Buchner O. Assessment of foliar frost damage: a comparison of in vivo chlorophyll fluorescence with other viability tests. J Appl Bot. 1999;73: 50–54.

[33] Taulavuori K, Taulavuori E, Sarjala T, Savonen EM, Pietilainen P, Lahdesmaki P, et al *In vivo* chlorophyll fluorescence is not always a good indicator of cold hardiness. J Plant Physiol. 2000;157: 227–229.

[34] Humphreys MW, Gąsior D, Leśniewska-Bocianowska A, Zwierzykowski Z, Rapacz M. Androgenesis as a means of dissecting complex genetic and physiological controls: selecting useful gene combinations for breeding freezing tolerant grasses. Euphytica 2007;158: 337–345.

[35] Sandve SR, Kosmala A, Rudi H, Fjellheim S, Rapacz M, Yamada T, Rognli OA. Molecular mechanisms underlying frost tolerance in perennial grasses adapted to cold climates. Plant Sci. 2011;180: 69–77. 10.1016/j.plantsci.2010.07.011 21421349

[36] Hüner NPA, Öquist G, Hurry V, Krol M, Falk S, Griffith M. Photosynthesis, photoinhibition and low temperature acclimation in cold tolerant plants. Photosyn Res. 1993;37: 19–39. 10.1007/BF02185436 24317651

[37] Rapacz M, Tyrka M, Kaczmarek W, Gut M, Wolanin B, Mikulski W. Photosynthetic acclimation to cold as a potential physiological marker of winter barley freezing tolerance assessed under variable winter environment. J Agr Crop Sci. 2008;194: 61–67.

[38] Rapacz M, Gąsior D, Zwierzykowski Z, Leśniewska-Bocianowska A, Humphreys MW, Gay AP. Changes in cold tolerance and the mechanisms of acclimatation of photosystem II to cold hardening generated be anther culture of *Festuca pratensis* × *Lolium multiflorum* cultivars. New Phytol. 2004;161: 105–114.

[39] Hömmo LM. Hardening of some winter wheat (*Triticum aestivum* L.), Triticale *(Triticosecale Wittmack)* and winter barley *(Hordeum vulgare L*.*)* cultivars during autumn and the final winter survival in Finland. Plant Breed. 1994;112: 285–293.

[40] Limin AE, Dvorak J, Fowler DB. Cold hardiness in hexaploid triticale. Can J Plant Sci. 1985;65: 487–490.

