# Supplementary figures and images for: Is the OJIP Test a Reliable Indicator of Winter Hardiness and Freezing Tolerance of Common Wheat and Triticale under Variable Winter Environments?
Source: PLoS One. 2015 Jul 31;10(7):e0134820. doi: 10.1371/journal.pone.0134820 (PMC4521754; doi:10.1371/journal.pone.0134820)

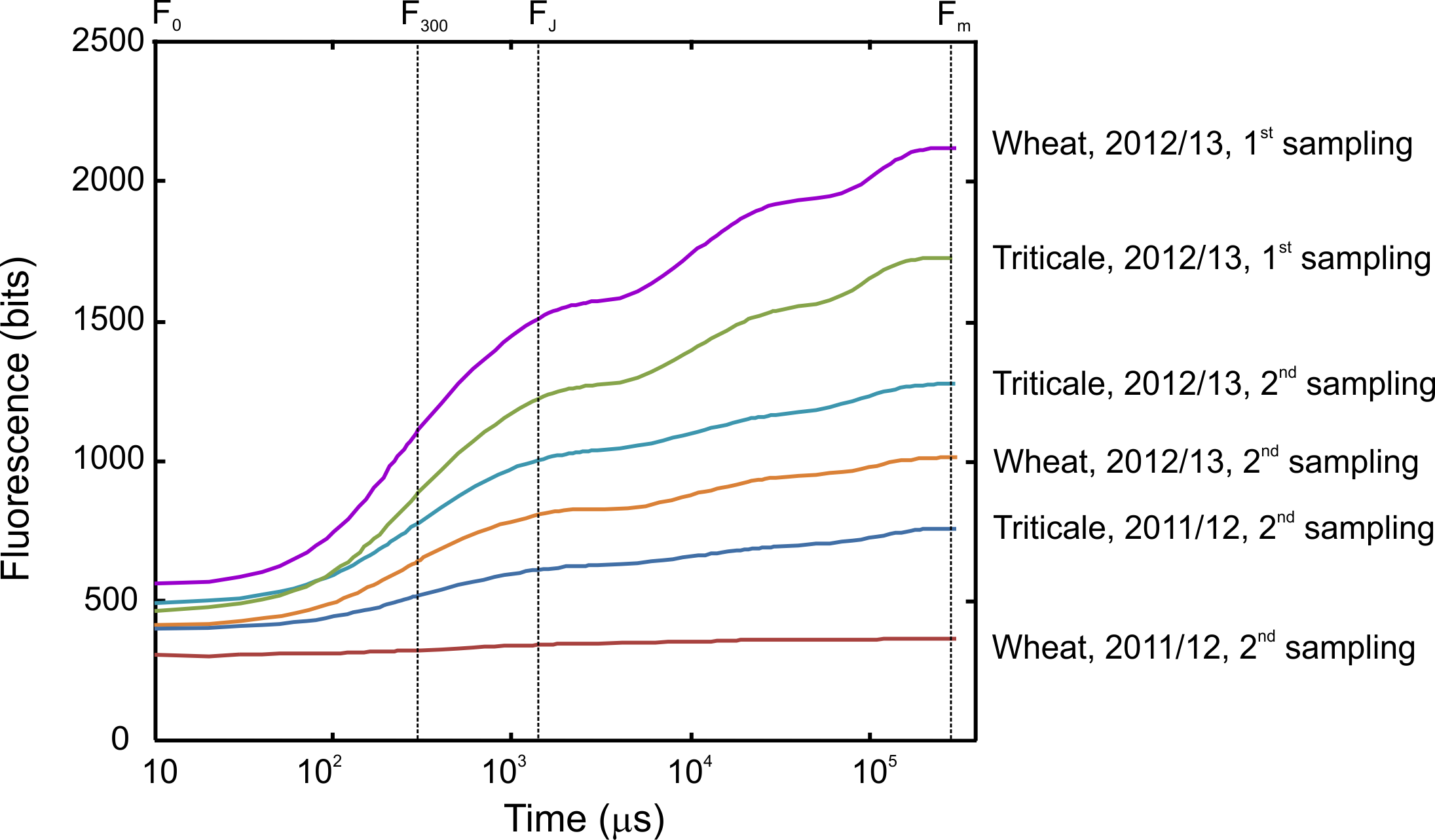

Supplement: S1 Fig — Time-points used for OJIP-test parameters calculation were indicated. (TIF) [file pone.0134820.s001.tif]
