# Supplementary material for: Is the OJIP Test a Reliable Indicator of Winter Hardiness and Freezing Tolerance of Common Wheat and Triticale under Variable Winter Environments?
Source: PLoS One. 2015 Jul 31;10(7):e0134820. doi: 10.1371/journal.pone.0134820 (PMC4521754; doi:10.1371/journal.pone.0134820)
Supplement: S1 Table — Asterisks are used to denote mean values that are statistically significantly different between wheat and triticale at P = 0.05. (DOCX) [file pone.0134820.s002.docx]

|  | Chlorophyll fluorescence parameter/winter and sampling | | | | | | | | | | | | | | | | | | | | | | | | | | | | | | |
| --- | --- | --- | --- | --- | --- | --- | --- | --- | --- | --- | --- | --- | --- | --- | --- | --- | --- | --- | --- | --- | --- | --- | --- | --- | --- | --- | --- | --- | --- | --- | --- |
| Winter (term)  Species | Fv/Fm | ABS/RC | ψo | | φEo | | PIcso | | PIcsm | | PI_ABS_ | | ABS/CS | | TRo/CS | | | ETo/CS | | DIo/CS | | RC/CSo | | RC/CSm | | ETo/RC | | TRo/RC | | | DIo/RC |
| 2010/11 (1) | | | | | | | | | | | | | | | | | | | | | | | | | | | | | | | |
|  |  | | | | | | | | | | | | | | | | | | | | | | | | | | | | | | |
| Wheat |  | | |  | |  | |  | |  | |  | |  | |  |  | |  | |  | |  | |  | |  | |  |  | |
| mean | 0.783 | 2.903 | 0.463^*^ | | 0.363^*^ | | 464.3^*^ | | 2191^*^ | | 1.109^*^ | | 419.5^*^ | | 328.5^*^ | | | 152.1^*^ | | 91.01 | | 144.8 | | 674.1 | | 1.050 | | 2.272^*^ | | | 0.630^*^ |
| min | 0.766 | 2.737 | 0.410 | | 0.318 | | 336.2 | | 1520 | | 0.859 | | 372.1 | | 286.2 | | | 129.1 | | 81.10 | | 126.0 | | 558.9 | | 0.904 | | 2.166 | | | 0.554 |
| max | 0.798 | 3.141 | 0.499 | | 0.395 | | 580.8 | | 2887 | | 1.395 | | 459.5 | | 358.9 | | | 166.2 | | 105.0 | | 160.8 | | 754.0 | | 1.130 | | 2.401 | | | 0.739 |
| Triticale |  |  |  | |  | |  | |  | |  | |  | |  | | |  | |  | |  | |  | |  | |  | | |  |
| mean | 0.765 | 3.145 | 0.386^*^ | | 0.296^*^ | | 317.5^*^ | | 1409^*^ | | 0.693^*^ | | 465.9^*^ | | 355.5^*^ | | | 136.9^*^ | | 110.4 | | 148.2 | | 640.0 | | 0.924 | | 2.399^*^ | | | 0.745^*^ |
| min | 0.730 | 2.983 | 0.326 | | 0.239 | | 209.4 | | 823.3 | | 0.430 | | 428.2 | | 329.0 | | | 119.5 | | 90.40 | | 135.2 | | 570.8 | | 0.795 | | 2.293 | | | 0.614 |
| max | 0.794 | 3.502 | 0.444 | | 0.349 | | 457.8 | | 2136 | | 0.974 | | 523.0 | | 380.6 | | | 164.1 | | 142.4 | | 155.6 | | 723.0 | | 1.054 | | 2.582 | | | 0.953 |
|  |  |  |  | |  | |  | |  | |  | |  | |  | | |  | |  | |  | |  | |  | |  | | |  |
| 2010/11 (2) | | | | | | | | | | | | | | | | | | | | | | | | | | | | | | | |
|  |  | | | | | | | | | | | | | | | | | | | | | | | | | | | | | | |
| Wheat |  | | |  | |  | |  | |  | |  | |  | |  |  | |  | |  | |  | |  | |  | |  |  | |
| mean | 0.463^*^ | 16.71^*^ | 0.251^*^ | | 0.135^*^ | | 86.43^*^ | | 286.8^*^ | | 0.150^*^ | | 666.1^*^ | | 297.9^*^ | | | 83.82^*^ | | 368.2^*^ | | 104.6^*^ | | 274.8^*^ | | 0.699^*^ | | 2.773 | | | 13.93^*^ |
| min | 0.188 | 3.807 | 0.095 | | 0.029 | | 4.687 | | 7.787 | | 0.007 | | 436.4 | | 131.0 | | | 21.07 | | 155.1 | | 46.09 | | 70.55 | | 0.295 | | 2.183 | | | 1.128 |
| max | 0.708 | 95.44 | 0.374 | | 0.266 | | 224.8 | | 855.0 | | 0.426 | | 885.2 | | 431.1 | | | 154.3 | | 665.0 | | 156.0 | | 499.2 | | 1.002 | | 3.138 | | | 93.03 |
| Triticale |  |  |  | |  | |  | |  | |  | |  | |  | | |  | |  | |  | |  | |  | |  | | |  |
| mean | 0.624^*^ | 7.032^*^ | 0.316^*^ | | 0.211^*^ | | 178.7^*^ | | 687.6^*^ | | 0.337^*^ | | 588.6^*^ | | 357.3^*^ | | | 119.0^*^ | | 231.3^*^ | | 132.9^*^ | | 442.1^*^ | | 0.844^*^ | | 2.667 | | | 4.365^*^ |
| min | 0.331 | 3.803 | 0.189 | | 0.070 | | 24.5 | | 79.29 | | 0.053 | | 461.5 | | 167.6 | | | 33.53 | | 157.7 | | 64.61 | | 140.5 | | 0.452 | | 2.318 | | | 1.091 |
| max | 0.724 | 36.24 | 0.394 | | 0.276 | | 308.7 | | 1227 | | 0.565 | | 738.8 | | 471.8 | | | 189.1 | | 450.4 | | 167.3 | | 604.2 | | 1.113 | | 2.932 | | | 33.66 |
| 2011/12 (1) | | | | | | | | | | | | | | | | | | | | | | | | | | | | | | | |
| Wheat |  | | |  | |  | |  | |  | |  | |  | |  |  | |  | |  | |  | |  | |  | |  |  | |
| mean | 0.810^*^ | 2.656^*^ | 0.522^*^ | | 0.424^*^ | | 893.6^*^ | | 4937^*^ | | 1.920^*^ | | 480.2^*^ | | 387.4 | | | 201.4 | | 92.7 | | 180.9 | | 968.8^*^ | | 1.116 | | 2.144 | | | 0.512^*^ |
| min | 0.757 | 2.459 | 0.439 | | 0.345 | | 571.2 | | 2757 | | 1.077 | | 403.2 | | 329.8 | | | 166.5 | | 69.9 | | 158.4 | | 781.6 | | 0.962 | | 2.003 | | | 0.410 |
| max | 0.837 | 3.248 | 0.590 | | 0.489 | | 1323 | | 7796 | | 2.822 | | 622.3 | | 477.9 | | | 231.6 | | 159.2 | | 202.7 | | 1120 | | 1.229 | | 2.391 | | | 0.856 |
| Triticale |  |  |  | |  | |  | |  | |  | |  | |  | | |  | |  | |  | |  | |  | |  | | |  |
| mean | 0.784^*^ | 2.987^*^ | 0.458^*^ | | 0.359^*^ | | 583.6^*^ | | 2841^*^ | | 1.120^*^ | | 538.4^*^ | | 419.9 | | | 192.1 | | 118.6 | | 179.9 | | 846.9^*^ | | 1.064 | | 2.331 | | | 0.656^*^ |
| min | 0.722 | 2.597 | 0.379 | | 0.274 | | 279.6 | | 1085 | | 0.539 | | 418.2 | | 344.0 | | | 146.9 | | 74.24 | | 145.2 | | 602.1 | | 0.914 | | 2.121 | | | 0.477 |
| max | 0.823 | 3.808 | 0.528 | | 0.430 | | 915.0 | | 4993 | | 1.858 | | 714.3 | | 525.3 | | | 237.0 | | 197.7 | | 204.4 | | 999.4 | | 1.192 | | 2.739 | | | 1.069 |
| 2011/12 (2) | | | | | | | | | | | | | | | | | | | | | | | | | | | | | | | |
| Wheat |  | | |  | |  | |  | |  | |  | |  | |  |  | |  | |  | |  | |  | |  | |  |  | |
| mean | 0.153^*^ | 66.98^*^ | 0.339 | | 0.057^*^ | | 12.55^*^ | | 28.22^*^ | | 0.036^*^ | | 308.3^*^ | | 50.04^*^ | | | 18.60^*^ | | 258.2 | | 27.64^*^ | | 46.47^*^ | | 0.772 | | 2.082 | | | 64.89^*^ |
| min | 0.030 | 9.900 | 0.215 | | 0.006 | | 0.03 | | 0.036 | | 0.000 | | 250.9 | | 9.440 | | | 2.097 | | 164.7 | | 6.274 | | 6.913 | | 0.333 | | 1.418 | | | 8.318 |
| max | 0.386 | 520.5 | 0.489 | | 0.149 | | 79.97 | | 230.8 | | 0.190 | | 396.5 | | 156.0 | | | 60.36 | | 324.2 | | 78.18 | | 197.6 | | 1.941 | | 4.834 | | | 515.6 |
| Triticale |  |  |  | |  | |  | |  | |  | |  | |  | | |  | |  | |  | |  | |  | |  | | |  |
| mean | 0.372^*^ | 21.64^*^ | 0.354 | | 0.138^*^ | | 64.36^*^ | | 176.9^*^ | | 0.146^*^ | | 400.2^*^ | | 160.3^*^ | | | 59.52^*^ | | 239.9 | | 75.42^*^ | | 176.7^*^ | | 0.758 | | 2.097 | | | 19.55^*^ |
| min | 0.077 | 3.978 | 0.232 | | 0.022 | | 0.746 | | 0.896 | | 0.002 | | 307.0 | | 25.78 | | | 7.708 | | 140.1 | | 15.09 | | 17.07 | | 0.477 | | 1.577 | | | 1.856 |
| max | 0.604 | 206.2 | 0.474 | | 0.249 | | 210.1 | | 685.9 | | 0.437 | | 601.8 | | 316.7 | | | 126.3 | | 381.5 | | 140.1 | | 434.7 | | 2.222 | | 4.069 | | | 202.2 |
| 2012/13 (1) | | | | | | | | | | | | | | | | | | | | | | | | | | | | | | | |
| Wheat |  | | |  | |  | |  | |  | |  | |  | |  |  | |  | |  | |  | |  | |  | |  |  | |
| mean | 0.754 | 3.087 | 0.347 | | 0.264 | | 309.2 | | 1380 | | 0.621^*^ | | 511.7^*^ | | 384.5^*^ | | | 133.2 | | 127.2 | | 167.3 | | 710.5^*^ | | 0.790 | | 0.792 | | | 2.295 |
| min | 0.696 | 2.668 | 0.259 | | 0.191 | | 176.0 | | 719.5 | | 0.341 | | 337.6 | | 257.8 | | | 96.3 | | 79.84 | | 121.7 | | 517.8 | | 0.606 | | 0.583 | | | 2.052 |
| max | 0.796 | 4.092 | 0.420 | | 0.329 | | 519.5 | | 2637 | | 1.137 | | 615.5 | | 447.0 | | | 169.6 | | 187.5 | | 194.2 | | 887.0 | | 0.949 | | 1.943 | | | 2.568 |
| Triticale |  |  |  | |  | |  | |  | |  | |  | |  | | |  | |  | |  | |  | |  | |  | | |  |
| mean | 0.746 | 2.941 | 0.382 | | 0.287 | | 309.9 | | 1372 | | 0.742^*^ | | 425.5^*^ | | 318.5^*^ | | | 121.6 | | 107.0 | | 147.2 | | 624.0^*^ | | 0.818 | | 0.789 | | | 2.152 |
| min | 0.640 | 2.625 | 0.333 | | 0.222 | | 187.1 | | 748.4 | | 0.439 | | 332.4 | | 236.5 | | | 93.4 | | 82.95 | | 121.4 | | 461.0 | | 0.699 | | 0.586 | | | 1.974 |
| max | 0.783 | 3.731 | 0.429 | | 0.329 | | 464.4 | | 2162 | | 0.983 | | 515.8 | | 398.6 | | | 164.1 | | 148.8 | | 183.1 | | 831.7 | | 0.893 | | 1.628 | | | 2.351 |
| 2012/13 (2) | | | | | | | | | | | | | | | | | | | | | | | | | | | | | | | |
| Wheat |  |  |  | |  | |  | |  | |  | |  | |  | | |  | |  | |  | |  | |  | |  | | |  |
| mean | 0.552 | 6.451 | 0.277 | | 0.168 | | 107.8 | | 407.4 | | 0.267 | | 421.8 | | 230.4 | | | 69.11 | | 191.4 | | 96.28 | | 298.5 | | 0.659 | | 4.008^*^ | | | 2.444 |
| min | 0.198 | 3.127 | 0.168 | | 0.037 | | 5.278 | | 11.06 | | 0.012 | | 320.0 | | 87.63 | | | 15.92 | | 94.23 | | 33.46 | | 52.94 | | 0.428 | | 0.836 | | | 2.018 |
| max | 0.744 | 20.01 | 0.385 | | 0.278 | | 261.1 | | 1081 | | 0.611 | | 554.6 | | 339.2 | | | 126.8 | | 356.7 | | 143.6 | | 562.4 | | 0.880 | | 17.41 | | | 2.773 |
| Triticale |  |  |  | |  | |  | |  | |  | |  | |  | | |  | |  | |  | |  | |  | |  | | |  |
| mean | 0.570 | 5.019 | 0.273 | | 0.168 | | 122.1 | | 434.7 | | 0.260 | | 476.1 | | 269.9 | | | 79.47 | | 206.1 | | 122.8 | | 367.0 | | 0.601 | | 2.802^*^ | | | 2.217 |
| min | 0.283 | 3.151 | 0.186 | | 0.057 | | 23.69 | | 71.44 | | 0.041 | | 370.5 | | 145.4 | | | 32.25 | | 135.3 | | 63.68 | | 124.5 | | 0.412 | | 0.972 | | | 1.846 |
| max | 0.700 | 11.57 | 0.364 | | 0.254 | | 232.4 | | 943.3 | | 0.526 | | 587.0 | | 371.6 | | | 123.2 | | 379.7 | | 165.8 | | 573.8 | | 0.790 | | 9.137 | | | 2.556 |
