# Supplementary material for: Is the OJIP Test a Reliable Indicator of Winter Hardiness and Freezing Tolerance of Common Wheat and Triticale under Variable Winter Environments?
Source: PLoS One. 2015 Jul 31;10(7):e0134820. doi: 10.1371/journal.pone.0134820 (PMC4521754; doi:10.1371/journal.pone.0134820)
Supplement: S2 Table — (DOCX) [file pone.0134820.s003.docx]

| Parameter | PC 1 | PC 2 | PC 3 | PC 4 | PC 5 | PC 6 |
| --- | --- | --- | --- | --- | --- | --- |
| Fv/Fm | 0.081 | 0.012 | 0.001 | 0.158 | 0.000 | 0.005 |
| ψo | 0.058 | 0.072 | 0.028 | 0.129 | 0.150 | 0.024 |
| φEo | 0.092 | 0.006 | 0.001 | 0.006 | 0.007 | 0.017 |
| PIcso | 0.078 | 0.034 | 0.021 | 0.030 | 0.067 | 0.023 |
| PIcsm | 0.072 | 0.047 | 0.021 | 0.042 | 0.106 | 0.063 |
| PI_ABS_ | 0.075 | 0.049 | 0.009 | 0.012 | 0.057 | 0.120 |
| ABS/RC | 0.033 | 0.067 | 0.351 | 0.012 | 0.021 | 0.009 |
| ETo/RC | 0.078 | 0.001 | 0.000 | 0.062 | 0.401 | 0.006 |
| TRo/RC | 0.003 | 0.258 | 0.072 | 0.018 | 0.057 | 0.386 |
| DIo/RC | 0.033 | 0.070 | 0.344 | 0.012 | 0.020 | 0.010 |
| ABS/CS | 0.008 | 0.241 | 0.074 | 0.140 | 0.012 | 0.064 |
| TRo/CS | 0.069 | 0.077 | 0.020 | 0.012 | 0.001 | 0.070 |
| ETo/CS | 0.090 | 0.003 | 0.024 | 0.008 | 0.011 | 0.031 |
| DIo/CS | 0.059 | 0.039 | 0.016 | 0.326 | 0.028 | 0.003 |
| RC/CSo | 0.083 | 0.022 | 0.006 | 0.019 | 0.025 | 0.140 |
| RC/CSm | 0.090 | 0.002 | 0.011 | 0.014 | 0.038 | 0.028 |
