# Supplementary material for: Is the OJIP Test a Reliable Indicator of Winter Hardiness and Freezing Tolerance of Common Wheat and Triticale under Variable Winter Environments?
Source: PLoS One. 2015 Jul 31;10(7):e0134820. doi: 10.1371/journal.pone.0134820 (PMC4521754; doi:10.1371/journal.pone.0134820)
Supplement: S1 Text — (DOCX) [file pone.0134820.s005.docx]

ABS / RC = M_0_ · (1 / V_J_) · (F_m_ / F_v_)

TR_o_ / RC = M_0_ · (1 / V_J_)

ET_o_ / RC = M_0_ · (1 / V_J_) · ψ_o_

DI_o_ / RC = (ABS / RC) – (TR_o_ / RC)

ABS / CS = F_0_

TR_o_ / CS = F_v_/F_m_ (ABS / CS_o_ )

ET_o_ / CS = F_v_/F_m_ · ψ_0_ · (ABS / CS_o_)

DI_o_ / CS = (ABS / CS) – (TR_o_/ CS)

RC / CS_o_ = F_v_/F_m_ · ( V_J_ / M_0_ ) · F_0_

RC / CS_m_ = F_v_/F_m_ · ( V_J_ / M_0_ ) · F_m_

PI_ABS_ = [1/(RC/ABS)] · [F_v_/F_m_/(1-F_v_/F_m_)] · [ψ_0_/(1–ψ_0_)]

PI_CSo_ = F_0_ · PI_ABS_

PI_CSm_ = F_m_ · PI_ABS_

where:

V_J_ = (F_J_ – F_0_) / (F_m_ – F_0_)

M_0_ = 4·(F_300_ – F_0_) / (F_m_ – F_0_)

F_v_ = F_m_ – F_0_

F_0_, F_300_, F_J_: chlorophyll fluorescence intensity after 10 μs, 300 μs and 2 ms of the recording. F_m_ – maximum fluorescence signal intensity
